# An earth observation and explainable machine learning approach for determining the drivers of invasive species — a water hyacinth case study

**DOI:** 10.1007/s10661-025-14517-1

**Published:** 2025-10-03

**Authors:** Geethen Singh, Benjamin Rosman, Marcus J Byrne, Chevonne Reynolds

**Affiliations:** 1 School of Animal, Plant & Environmental Sciences, University of the Witwatersrand, Johannesburg, South Africa; 2 Fitzpatrick Institute of African Ornithology, DST‐NRF Centre of Excellence,Department of Botany and Zoology, Centre for Invasion Biology , University of Cape Town, Stellenbosch University, RondeboschStellenbosch, South Africa; 3 Department of Botany and Zoology, Centre for Invasion Biology, Stellenbosch University, Stellenbosch, South Africa; 4 School of Computer Science & Applied Mathematics, University of the Witwatersrand, Johannesburg, South Africa; 5 Machine Intelligence Neural Discovery (MIND) Institute, University of the Witwatersrand, Johannesburg, South Africa; 6 Centre for Invasion Biology, School of Animal, Plant and Environmental Sciences, University of the Witwatersrand, Johannesburg, South Africa

**Keywords:** Satellite, Remote sensing, Google Earth Engine, Explainable artificial intelligence, Habitat suitability, Biological invasions

## Abstract

Invasive species management is often constrained by limited resources and complicated by ecological and socio-economic variability across landscapes, leading to inconsistent outcomes. We use water hyacinth (*Pontederia crassipes*) in South Africa as a case study to demonstrate how combining earth observation (EO) data, species distribution models (SDMs), and explainable artificial intelligence (xAI) can support more spatially explicit and context-sensitive management strategies. Despite decades of control efforts, water hyacinth remains widespread, with its proliferation shaped by ecological and socio-economic contexts in which the weed proliferates. Using SHapley Additive exPlanations (SHAP), we studied the environmental and socio-economic contexts impacting water hyacinth prevalence across multiple spatial scales in South Africa. Consistent patterns emerged with known physiological constraints, such as minimum temperature, while novel spatial trends were revealed—highlighting temperature effects along the coast and the role of vegetation type in inland regions. These insights offer opportunities for targeted fieldwork to investigate emergent non-linear relationships and interaction effects between covariates. The spatially explicit outputs, covering all South African water bodies, provide a low-cost, scalable tool to guide the prioritization of risk, inform monitoring and early detection efforts, and support the selection of locally appropriate management strategies. While focused on water hyacinth, our approach is generalizable to other invasive species, illustrating the value of integrating EO data and xAI to enhance understanding of species-environment dynamics and enable adaptive, data-driven intervention planning.

## Introduction

Biological invasions impose significant economic, ecological, and societal costs (Cuthbert et al., 2022; Diagne et al., 2020). Species distribution models (SDMs) are widely used to estimate the potential distribution of invasive species within their non-native range and under projected climate change scenarios (Barbet-Massin et al., 2018; Elith, 2017). While machine learning models are frequently employed for this purpose (Elith, 2017), they are often discouraged when the primary objective is to understand the mechanistic relationships between an invasive species and its biotic or abiotic environment, due to their tendency to function as black-box models that typically exhibit good predictive performance but with a trade-off in interpretability. To address this limitation, the field of explainable artificial intelligence (xAI), which focuses on making the inner workings and decision processes of complex models more transparent, has introduced two widely used post hoc tools: feature importance and partial dependence plots (Ryo et al., 2021). Feature importance attributes the relative importance of covariates in discerning species presence from absence, while partial dependency plots elucidate the relationship between a covariate and the probability of a species occurrence. Despite the promise of these tools, both largely ignore the spatial dimension, leading to analyses that largely exclude the role of location and context (Roussel & Böhm, 2023).

Incorporating spatially explicit information on how invasive species respond to environmental, socio-economic, and ecological factors across different locations would greatly enhance the selection of management interventions, guide efficient resource allocation, and improve risk-management strategies (McGeoch et al., 2016). Resource allocation for weed control in invasive species management is complex, requiring strategic distribution of limited resources across multiple invasive species, extensive areas, diverse management jurisdictions, and various management strategies, often with limited information on their context-dependent effectiveness (Baker, 2017). A shift toward pre-emptive management requires understanding the ecological and socio-economic contexts influencing the risk of invasive species establishment and spread (John R Wilson et al., 2005). Understanding where and why invasive species occur can inform management planning by identifying (1) locations where specific interventions may be most appropriate, (2) areas unsuitable for current approaches that could be used to develop and test new management strategies, and (3) sites with high susceptibility to invasion that should be prioritized for early detection and monitoring efforts. Although our study does not incorporate data on management history during modeling, these speculative cases highlight the potential value of spatially explicit species-environment relationships to support intervention type, prioritization, and monitoring efforts (King, 2011; Rainford et al., 2020).

While monitoring provides insights into new weed infestations and the actual alien plant’s distribution (Kilroy et al., 2008), modeling habitat suitability and susceptibility offers valuable predictions of the risk of future invasions. SDMs address this need by representing the relative likelihood of an alien plant establishing should the species be introduced to each location in the modeled landscape (Barbet-Massin et al., 2018; Briscoe Runquist et al., 2021; Elith, 2017). To model the combined risk of susceptibility and suitability, SDMs have been extended to include features that influence the introduction and spread of species (e.g., distance to roads, and the presence/absence of other species as proxies for biotic interaction) (Kumar et al., 2014; Srivastava et al., 2019; Wisz et al., 2013). These enhanced models provide a more comprehensive understanding of invasion risk, integrating both environmental suitability and potential pathways for species spread.

While a comprehensive Risk Analysis for Alien Taxon (RAAT) has been established (Kumschick et al., 2020), it is not suited to spatially explicit and fine-scaled risk quantification. Previous studies investigating the risk of invasive plant distribution shifts under varying climate change scenarios have utilized mechanistic climate matching models (CLIMEX) (Kriticos & Brunel, 2016) and correlative SDMs (Kriticos & Brunel, 2016; Rodriguez-Merino et al., 2018). However, several challenges limit the applicability of previous results, including the use of a restricted set of features during modeling (Ahmed et al., 2020; Elith & Leathwick, 2009), reliance on presence-only data, and insufficient attention to spatial autocorrelation and spatially explicit analyses of feature importance during model selection, feature selection, and model interpretability (Domisch et al., 2019; Elith & Leathwick, 2009). The present study directly addresses all of these limitations by incorporating presence-absence data based on a pre-existing satellite-derived distribution of water hyacinth and a broader set of EO-derived datasets for predictors (Singh et al., 2020), accounting for spatial structure in the data, while applying spatially explicit techniques to interpret model outputs captured by an SDM.

Unlike field-based approaches, earth observation (EO) data—when combined with recent advances in explainable AI (xAI)—are well-suited for SDMs across large areas and multiple spatial scales (Cha et al., 2021). This advantage can be attributed to free EO data policies along with the global, systematic, and frequent acquisition of these data (e.g., Grill et al., 2019; Kennedy et al., 2019; Pekel et al., 2016). These developments have encouraged the creation of EO-derived data products that capture ecological, hydrological, climatological, social, and topographical phenomena across the Earth’s surface (Bradie & Leung, 2017; Braunisch et al., 2013). Furthermore, the recent availability of free cloud computing infrastructure and open-source libraries has significantly lowered the barriers to large-scale EO data analysis (Jordahl, 2014). This combination of accessible data and tools enables researchers to conduct comprehensive, wide-ranging studies of invasive species distributions with unprecedented detail and scale.

As a case study, we focus on water hyacinth *Pontederia* (previously *Eichhornia*) *crassipes* (Pellegrini et al., 2018), which is listed among the “100 of the world’s worst” invasive species by the IUCN. It is recognized as the most problematic Invasive Alien Aquatic Plant (IAAP) species in terms of its impacts and difficulty to manage (Vila & Ibáñez, 2011; Villamagna & Murphy, 2010). While water hyacinth was first recorded in South Africa in 1908, it has been the target of extensive management efforts since the 1960 s (Bick et al., 2020; J A Coetzee et al., 2011; Miller et al., 2021; Tipping et al., 2020). Despite many efforts to eradicate and/or control the weed, water hyacinth proliferates and was estimated to cover 417.7 km^2^ of South Africa during 2013 (Singh et al., 2020). The inconsistent success in managing water hyacinth has been attributed to abiotic factors including low temperature, wind conditions, excess water nutrient conditions, and the largely reactive nature of biological, mechanical, and chemical management strategies (Martin P Hill & Coetzee, 2017).

This study demonstrates, for the first time, the utility of integrating a machine learning-based species distribution modeling (SDM) approach with a post hoc explainable AI tool—SHapley Additive exPlanations (SHAP)—using EO-derived data products and cloud computing resources available through Google Earth Engine (GEE) to (1) determine the relative importance of biotic and abiotic factors likely influencing the occurrence of water hyacinth across multiple spatial scales, from individual water bodies to provincial and national extents; (2) capture the relationship between socio-economic and environmental factors and the probability of water hyacinth occurrence; and (3) elucidate the influence of estimated interaction effects between variables on the probability of water hyacinth occurrence. Together, these contributions represent a low-cost, desktop-based method that provides spatially explicit information to support and guide management strategy selection, resource allocation, and risk management —an approach that is transferable to other invasive alien plant species.

## Materials and methods

**Fig. 1:**
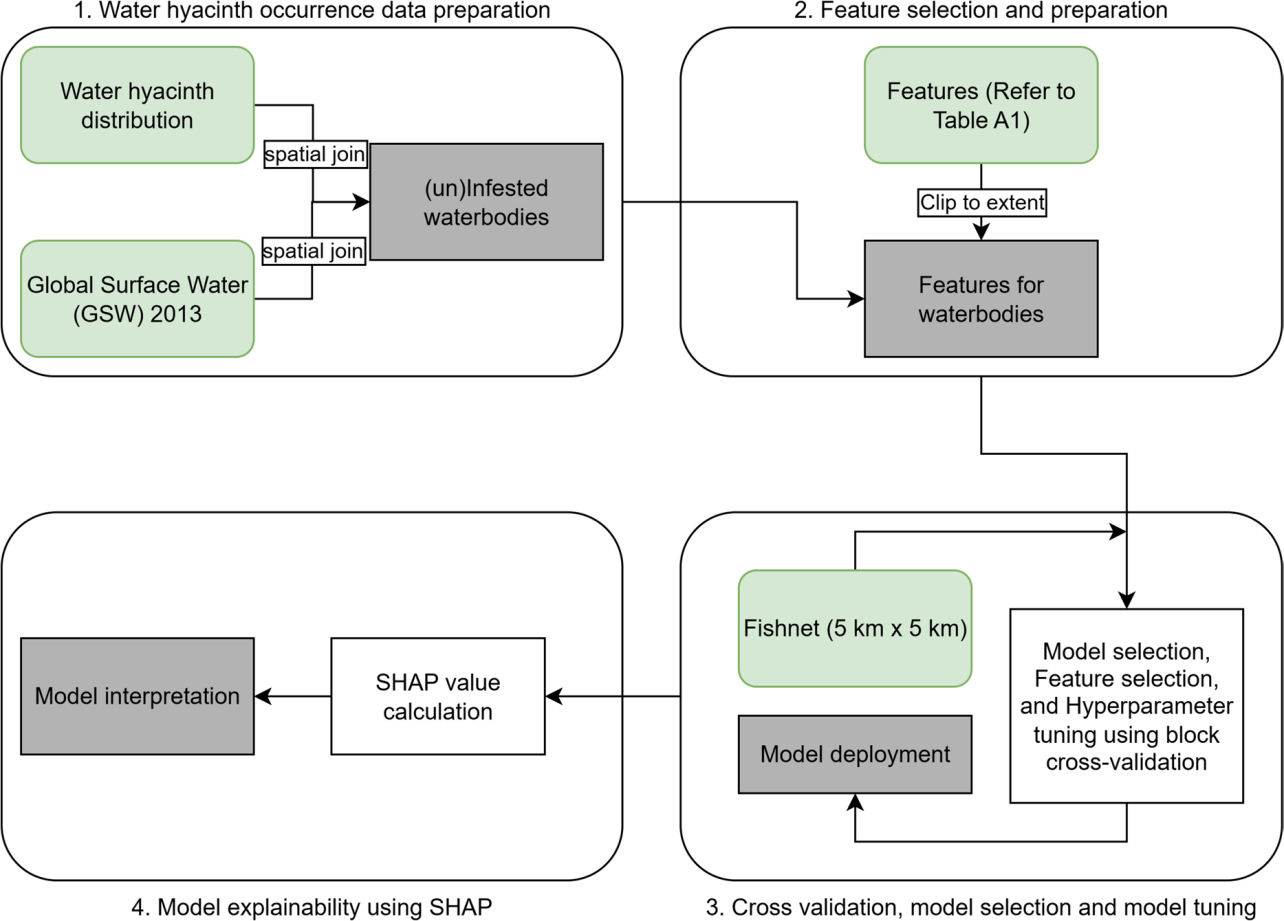
The general workflow used in this study to determine the relative importance of likely drivers of water hyacinth occurrence. The workflow highlights three main types of components: inputs (green) pre-processing and modeling processes (white), and outputs (gray)

This study followed four distinct phases (Fig. 1). These include (1) preparation of water hyacinth occurrence data; (2) selection and preparation of pertinent features; (3) fine-tuning of the model parameters coupled with cross-validation; and (4) model explainability.

### Water hyacinth occurrence data preparation

To support large-scale species distribution modeling, we used a satellite-derived presence–absence map of water hyacinth as a spatially consistent and cost-effective alternative to field-based surveys. Unlike presence-only data, which are limited by uncertain absences, prevalence distortion, and sampling bias, presence–absence data allow the application of balanced classification metrics. This includes the Matthews correlation coefficient (MCC) and F1-score that are robust to class imbalance—where the number of invaded water bodies (positives) is much smaller than the number of uninvaded ones (negatives)— and enable meaningful comparisons across time periods, regions, and species. Although satellite-derived presence–absence labels may contain classification errors, we adopted conservative model selection and hyperparameter tuning strategies (described later) to reduce their influence.

**Fig. 2:**
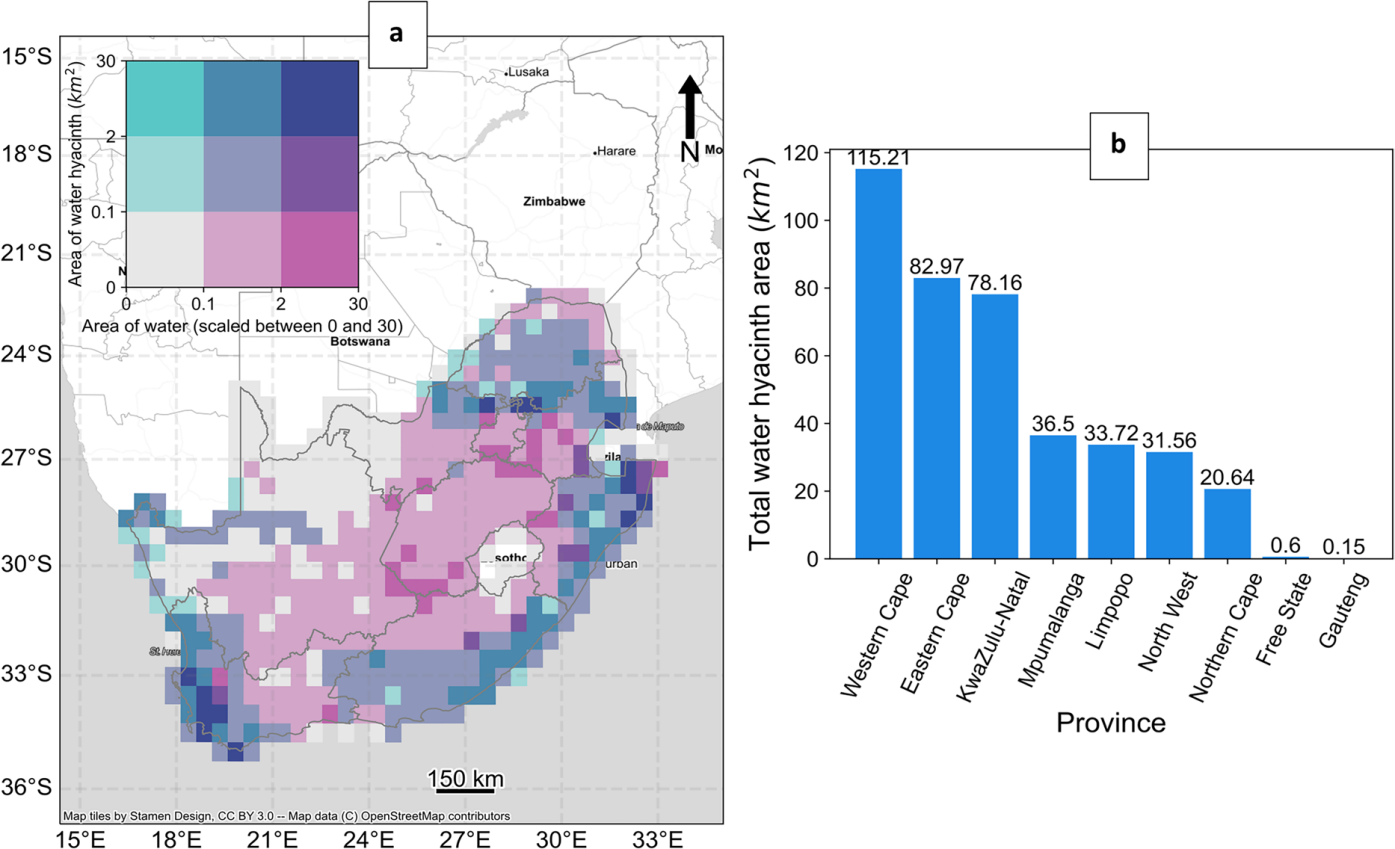
(**a**) Distribution of surface water area (km², source: Global Surface Water 2013) and the area of water hyacinth across South Africa (source: Singh et al., 2020), aggregated at 1°, and (**b**) per province, showing the Western Cape, Eastern Cape, and KwaZulu-Natal provinces as the most affected regions. Dark blue areas correspond to the largest water hyacinth infestations in the country. The inset legend shows the joint distribution of surface water area and water hyacinth area. Each color represents a unique combination of water hyacinth area (vertical axis, in km²) and total surface water area (horizontal axis, scaled between 0 and 30 km²) per grid cell. Darker colors (e.g., purple) indicate areas with both extensive surface water and high water hyacinth abundance. The original fine-scale (30 m) distribution of water hyacinth can be viewed in the Appendix, Fig. 9

The national water hyacinth distribution map was generated using a remote sensing approach developed by Singh et al. (2020). Their method involved a three-stage process: first, surface water bodies were mapped using satellite-derived water indices; second, aquatic vegetation was identified within these areas using thresholding and segmentation techniques; and third, water hyacinth was distinguished from other aquatic vegetation types using a supervised classification model trained on a combination of 98 in-field survey sites and environmental predictors, including topographical, climatic, and meteorological variables, as well as Landsat-8 spectral reflectance. For each site, all pixels containing aquatic vegetation were used to train a classification model at 30-m spatial resolution. The resulting map estimated that water hyacinth covered 2.69% of the total surface water area in South Africa in 2013, with a classification accuracy of 80% based on the MCC (Appendix, Fig. 9). Areas with the highest infestation—reflecting both high hyacinth abundance and environmentally suitable conditions—were located in the Western Cape province, northeastern KwaZulu-Natal province, and along the northern boundary of the Gauteng province (Fig. 2).

Given the limited number of high-quality, water body-level field observations (*n* = 98) relative to the large number of candidate environmental predictors (*n* = 140), we used the satellite-derived distribution from Singh et al. (2020) to generate presence–absence labels suitable for model training. This decision was motivated by two factors. First, relying solely on the 98 water bodies would have increased the risk of overfitting, compromising model generalizability (Johnstone & Titterington, 2009; Kwon & Sim, 2013). Second, the original classification was performed at the pixel level using reflectance data, whereas our modeling framework required covariates aggregated at the water body scale.

To ensure compatibility, we converted the predicted distribution to water body-level presence–absence labels by spatially joining the vectorised hyacinth map with the Global Surface Water dataset (Pekel et al., 2016). Water bodies with any predicted hyacinth presence were classified as positive (*n* = 27,206), while those without were labelled negative (*n* = 221,164). This process yielded a spatially extensive dataset appropriate for large-scale species distribution modeling and downstream analyses.

### Feature selection and preparation

We considered a total of 140 features for this study (Table A1), selecting them based on their availability as Earth Observation (EO) data products for South Africa and their documented influence on water hyacinth. In addition to considering fine-class land cover (73 of 140 variables) and broad-class land cover (10 of 140) (GEOTERRAIMAGE, 2020), we also considered 57 other climatic (e.g., temperature, precipitation, frost and wind), socio-economic (e.g., relative wealth index, human modification index), ecological and hydrological features (e.g., interspecies competition, runoff, flood risk and riparian soil nutrients, Table A2). In instances where variables were not available for the year 2013, the closest temporal data was utilized (21 variables). Only data from 1970 to 2019 were used in the analysis, with the exception of the soil organic carbon layer from SoilGrids, which includes fewer than 5% of in situ measurements collected prior to 1960 (Poggio et al., 2021). For datasets not readily available within the Google Earth Engine (GEE) data catalogue or GEE community datasets (Roy et al., 2023), datasets were downloaded (Table A1) and subsequently uploaded to GEE for analysis.

We extracted water body-level covariates by summarizing values for each vectorized surface water polygon from the 2013 Global Surface Water dataset (Pekel et al., 2016). For variables natively available at 30-m resolution—such as minimum temperature, elevation, and vegetation indices (e.g., EVI)—we computed the mean value across all pixels intersecting each water body. Datasets with coarser native resolution were resampled to 30 m using bilinear interpolation within GEE, which automatically performs this resampling when computing zonal statistics at a specified scale. Since covariates were ultimately aggregated at the water body level, this resampling step was a technical convenience rather than a transformation of pixel-level data used directly in modeling. For landscape-context variables—including soil properties, flood risk, and nutrient levels—we applied a 5-km buffer around each water body and extracted mean values. For land cover, we calculated class-specific area totals within the buffer, avoiding interpolation due to the categorical nature of the data. For area calculations, GEE internally reprojects datasets to an equal-area projection determined by the region of interest. All other geoprocessing used the default EPSG:4326 (WGS84) as the working coordinate reference system. All input layers were provided in standard geospatial formats (GeoTIFF for rasters, shapefile for vectors); no non-spatial tabular formats (e.g., CSV or TXT) were used. The covariates extracted at the polygon or buffer scale served as water body-level summaries for subsequent modeling.

Given the extensive array of environmental layers (*n* = 140), we implemented a reproducible procedure to select features for modeling. Feature selection is beneficial because it (1) mitigates redundancy among features; (2) promotes model parsimony and computational efficiency; (3) reduces the risk of overfitting; (4) precludes a biased evaluation of feature importance; (5) mitigates the adverse impact of high dimensionality on model performance; and (6) simplifies model interpretation (Chandrashekar & Sahin, 2014; Guyon & Elisseeff, 2003). Our feature selection procedure consisted of three sequential steps: first, we excluded unsuitable features based on low variance; next, we removed redundant features by analyzing their correlation with the remaining features; and finally, we eliminated features with low predictive power.

#### Step 1: Removal of irrelevant features

We excluded variables related to the count of consecutive nights with temperatures below 10 °C and the quantity of upstream rivers due to their low variance across water bodies, indicating limited predictive power in modeling. Additionally, we removed irrelevant features, such as snow and moss cover fraction derived from global, broad-class landcover data (Buchhorn et al., 2020), as these variables have limited relevance in the South African context.

#### Step 2: Selection of uncorrelated variables

To mitigate redundancy among the remaining 136 layers, we conducted a selection of uncorrelated variables by retaining only those features with an absolute pairwise correlation coefficient less than |0.7| with all other features for subsequent analyses (Dormann et al., 2013). When a pair of features exceeded this correlation threshold, we manually selected the most appropriate feature. This manual selection enabled us to prioritize features with higher spatio-temporal resolution, future scalability, and global availability. For example, minimum temperature in the coldest month was sourced from the WorldClim v1 dataset (1970–1990), which was the most recent version available in GEE at the time of analysis. Despite the temporal mismatch with our 2013 baseline, we retained this layer due to its widespread use, full spatial coverage, and strong correlation with more recent MODIS-derived cold season metrics. The decision reflected a trade-off between temporal precision and practical considerations such as data availability and processing efficiency. While our study used the most accessible climatic and environmental predictors available in GEE at the time, we acknowledge that newer high-resolution datasets, such as CHELSA V2.1, may offer improved accuracy and relevance for more recent timeframes. Future efforts should consider integrating such updated sources, particularly for applications requiring contemporary climate baselines. This stage reduced the feature set to 103 variables, which we then subjected to the final stage of selection.

#### Step 3: Recursive feature elimination

The recursive feature elimination with cross-validation (RFECV) method we implemented effectively selects optimal feature sets for both tree-based and linear models (e.g. Gomes et al., 2019; Pullanagari et al., 2018). In our case, the algorithm initiates by fitting a random forest model with all 103 features, evaluating performance, and ranking feature importance. It then iteratively removes the least important features, re-fits the model, and evaluates it, continuing this process until identifying an optimal set of covariates that do not decrease the F1 score. RFECV produced a final selection of 82 features for modeling (Table A1). We chose RFECV over alternatives like elastic net regularization because of its suitability for non-linear problems and its compatibility with a block Cross-Validation (CV) approach, which accounts for spatial autocorrelation (Kuhn & Johnson, 2013; Roberts et al., 2017).

### Cross-validation (CV), model selection, and model tuning

To obtain realistic and generalizable performance estimates, it is essential to evaluate models using cross-validation. However, when applied to spatial data, conventional random k-fold cross-validation can lead to overly optimistic results, as the spatial autocorrelation between training and test data allows models to exploit spatial proximity rather than learning true ecological relationships (Roberts et al., 2017). Moreover, spatial variability in the density of invaded and uninvaded water bodies can result in an uneven distribution of classes across folds, further biasing performance estimates. Therefore, careful spatial partitioning is necessary to ensure that each fold represents a spatially independent and balanced subset of the data. This, in turn, avoids class imbalance and inflated spatial autocorrelation within folds, which may lead to artificially high model accuracy (Meyer et al., 2019; Ploton et al., 2020; Valavi et al., 2019). To address these issues, we adopted a block cross-validation (CV) strategy, spatially aggregating presence/absence observations into 1° (∼111 km) blocks. This block size was selected for practical and computational reasons, rather than being based on a specific ecological process such as organismal dispersal distance. We then randomly assigned these blocks to one of ten validation folds, ensuring that all instances, both presences and absences, within a block remained in the same fold (either training or validation, but not both) throughout model calibration and validation. This approach ensured a more balanced distribution between positive and negative classes and reduced spatial autocorrelation within each fold. During tenfold block CV, the model trained iteratively on nine folds, with validation on the remaining fold, repeating this process until each fold served as a validation set, collectively referred to as block CV. This approach differs from spatial thinning (e.g., via the spThin package (Aiello‐Lammens et al. 2015)), which discards spatially proximate records, whereas block CV retains all records but partitions them into spatially structured folds to control for spatial dependence during model evaluation. We applied block CV for model selection, feature selection, hyperparameter tuning, model evaluation, and model interpretation.

For assessing models fitted to imbalanced datasets—where the number of invaded water bodies (positives) is much smaller than the number of uninvaded ones (negatives)—we used precision, recall, F1-score, Matthews correlation Coefficient (MCC), and balanced accuracy as evaluation metrics. These metrics are particularly advantageous due to their reduced sensitivity to variations in the number of positive and negative instances (Chicco & Jurman, 2020). All metrics range from 0 to 1, except for MCC, which ranges from − 1 to 1, with higher values indicating better model performance.

Precision measures the ratio of true positives (correctly classified water hyacinth infestations) to total predicted positives (both correctly and incorrectly classified infestations). Recall represents the ratio of true positives to all actual positives (correctly classified infestations and misclassified uninvaded water bodies). Precision is prioritized when the cost of misclassifying uninvaded water bodies as invaded (i.e., false positives) is high, while recall is prioritized when the cost of falsely classifying actual infestations as uninvaded (i.e., false negatives) is greater. In this study, we optimized the precision score during model selection and hyperparameter tuning. This decision aimed to reduce the effect of false positives originating from the satellite-derived distribution used to define water hyacinth presence. Since the primary objective of our analysis was to generate interpretable models (rather than operational detection), minimizing false associations between covariates and incorrectly labeled positive instances was critical. High precision ensures that the model is less likely to incorrectly identify a water body as infested, which improves the reliability of subsequent interpretability analyses, such as SHAP-based feature attribution. However, if the model were to be used for risk mapping or surveillance planning, where the priority is to avoid overlooking actual infestations, then recall (sensitivity) would be a more suitable optimization target. In such cases, the model would prioritize capturing as many true infestations as possible, even at the cost of more false positives. When both objectives—accurate explanation and robust detection—are equally important, F1-score, which balances precision and recall, may offer a suitable compromise.

**Fig. 3:**
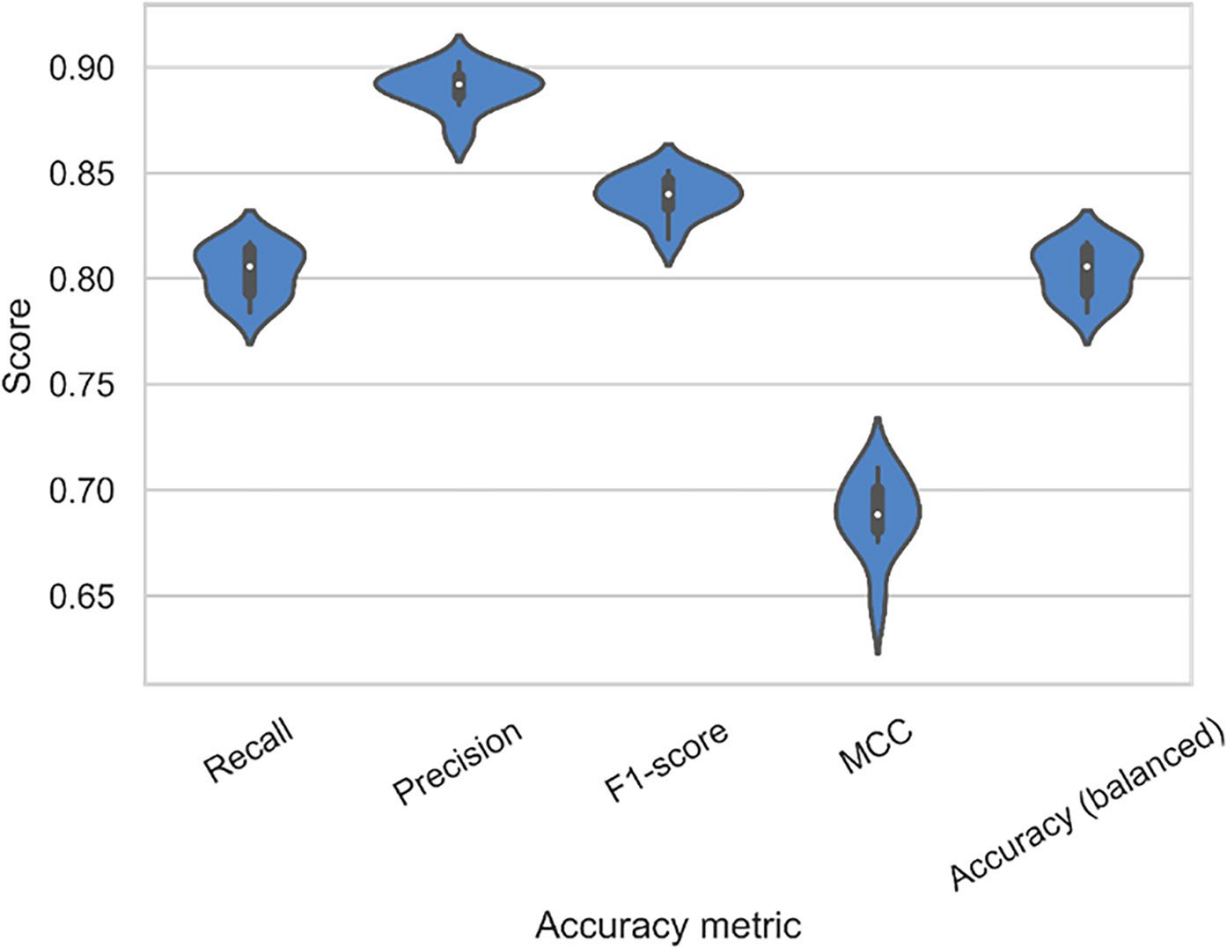
The distribution (blue) and box plots of the random forest model evaluation metrics, post-hyperparameter optimization. Scores are based on ten fold block cross-validation. The metrics include precision, recall, F1-score, and the Matthews correlation coefficient (MCC)

In the model selection stage, we evaluated 15 candidate machine learning classifiers implemented in the PyCaret Python package. These included ensemble-based decision tree models (e.g., random forest, extra trees, gradient boosting, AdaBoost, CatBoost, and extreme gradient boosting), linear models (e.g., logistic regression, ridge classifier, linear and quadratic discriminant analysis), support vector machines (SVM with a linear kernel), k-nearest neighbors, and probabilistic models such as Naive Bayes. The performance of each model was assessed using multiple metrics (e.g., F1-score, MCC, precision, recall; see Table A3), leading to the selection of the random forest classifier as the optimal model based on mean precision score (Table A3 and Fig. 3). The random forest algorithm constructs an accurate classifier by aggregating multiple weak classifiers, specifically decision trees (Breiman, 2001). The ensemble-based random forest algorithm randomly selects multiple subsets of explanatory variables to train distinct decision tree models. Each tree in the ensemble independently predicts whether a water hyacinth infestation exists within a water body, and the final prediction is based on the aggregated votes of all trees. Random forests have gained widespread use in species distribution modeling due to their robustness to overfitting, ability to handle nonlinear relationships, and effectiveness with high-dimensional data (Cutler et al., 2007; Mi et al., 2017).

We optimized the hyperparameters of the random forest model using sequential model-based optimization (SMBO), a computationally efficient alternative to exhaustive grid or random search methods (Wistuba et al., 2015). SMBO iteratively identifies promising hyperparameter combinations based on expected improvements in model performance, reducing the number of evaluations required. The tuning process considered the number of trees in the forest, the maximum tree depth, the minimum number of samples required to split an internal node, the minimum number of samples required at a leaf node, and whether or not bootstrap sampling was used. The final selected values were 542 trees, no maximum depth, a minimum of 2 samples to split a node, a minimum of 1 sample at each leaf, and no bootstrap sampling. These values were chosen to maximize model performance on the training data and are not intended to generalize across different study systems.

### Model explainability using SHAP

Model explainability was used to understand the drivers of water hyacinth occurrence across South Africa. In this study, we use SHapley Additive exPlanations (SHAP) to quantify each feature’s contribution to the model’s predictions. SHAP was selected for its theoretical rigor and its flexibility in providing both local and global interpretability.

#### Overview of SHAP

SHAP is a post hoc interpretability tool that can be combined with any machine learning model commonly used in SDMs. The contributions of a feature are computed by considering how the inclusion or exclusion of a feature changes the average model output, in our case the probability of water hyacinth occurrence at a water body (Lundberg et al. 2020). SHAP is preferred and increasingly adopted for model interpretation across various domains (Roscher et al., 2020), including ecology (e.g., Cha et al., 2021; Wang et al., 2021; Yu et al., 2020), because of its theoretical justification (e.g., additivity and consistency properties) and analytical advantages over other proposed xAI tools (e.g., local interpretable model-agnostic explanations (LIME) and mean decrease in impurity (MDI) feature importance). SHAP ensures consistency between local and global interpretations by guaranteeing that the sum of a feature’s contributions across all individual predictions matches its overall importance. This means features that have greater influence locally will also be ranked higher globally, making SHAP a reliable tool for both levels of model interpretation.

#### SHAP analysis for water hyacinth occurrence

**Fig. 4:**
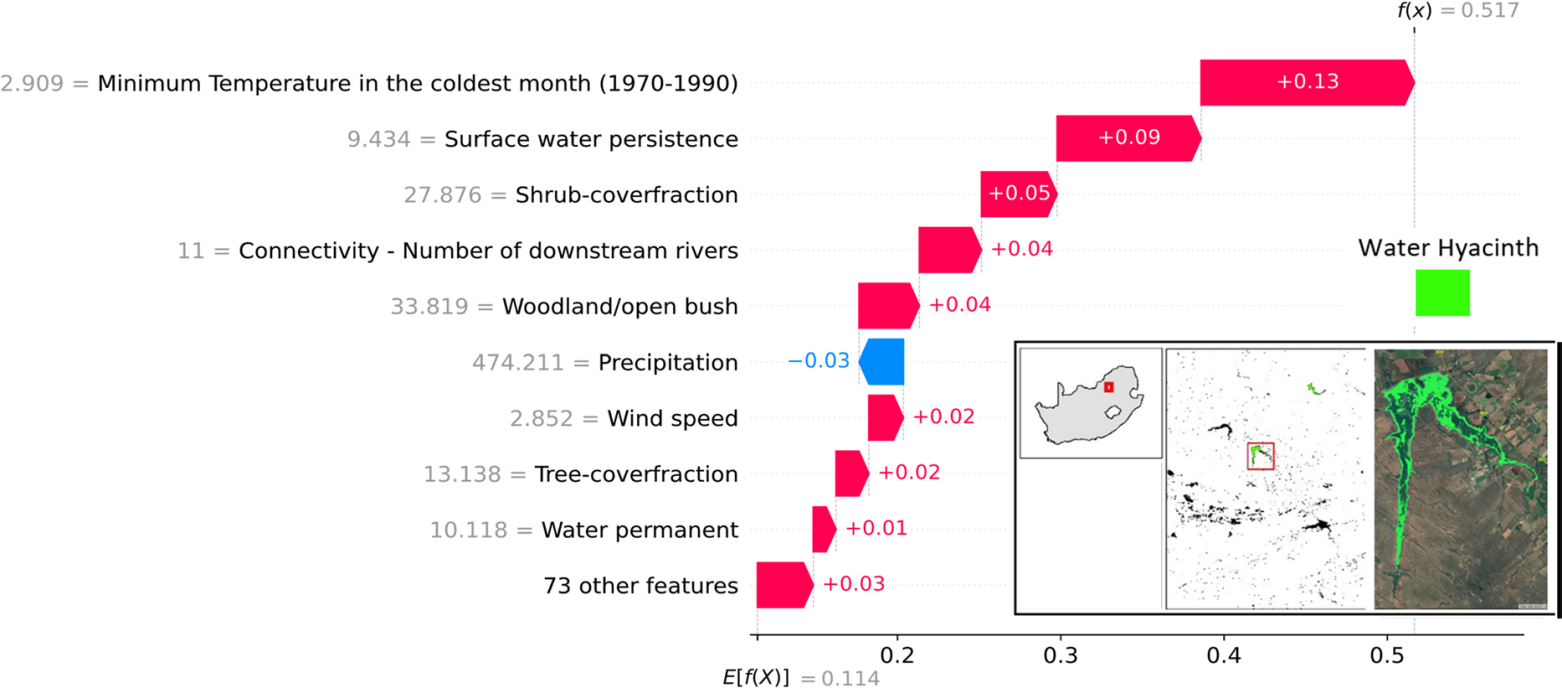
SHAP summary plot showing the top predictors influencing the modeled probability of water hyacinth presence at Roodekoppies Dam, South Africa. The actual feature values for this dam are shown in gray to the left of each predictor label. The colored bars indicate the contribution (SHAP value) of each feature to the site-specific prediction (*f(X)* = 0.517) relative to the average prediction across all sites (*E[f(X)]* = 0.114). Features that increase site suitability (positive SHAP values) are shown in red, while those that reduce it (negative SHAP values) appear in blue. For instance, a relatively warm minimum temperature in the coldest month (2.909 °C) has the greatest positive contribution (+ 0.13), while near-average precipitation (474.21 mm) slightly decreases site suitability (–0.03). Inset maps show the dam’s location, and the 2013 accumulated water hyacinth cover is shown in green

**Fig. 5:**
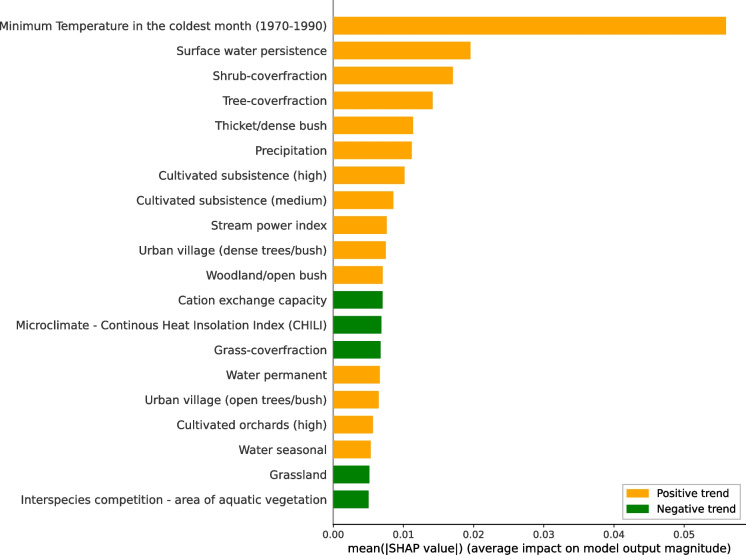
Feature importance for the top 20 features used to classify the occurrence of water hyacinth, sorted by their mean contribution (absolute SHAP values) to predict water hyacinth presence across South Africa for 2013. Features show either a positive (orange) or a negative effect (green) on the probability of water hyacinth occurrence

**Fig. 6:**
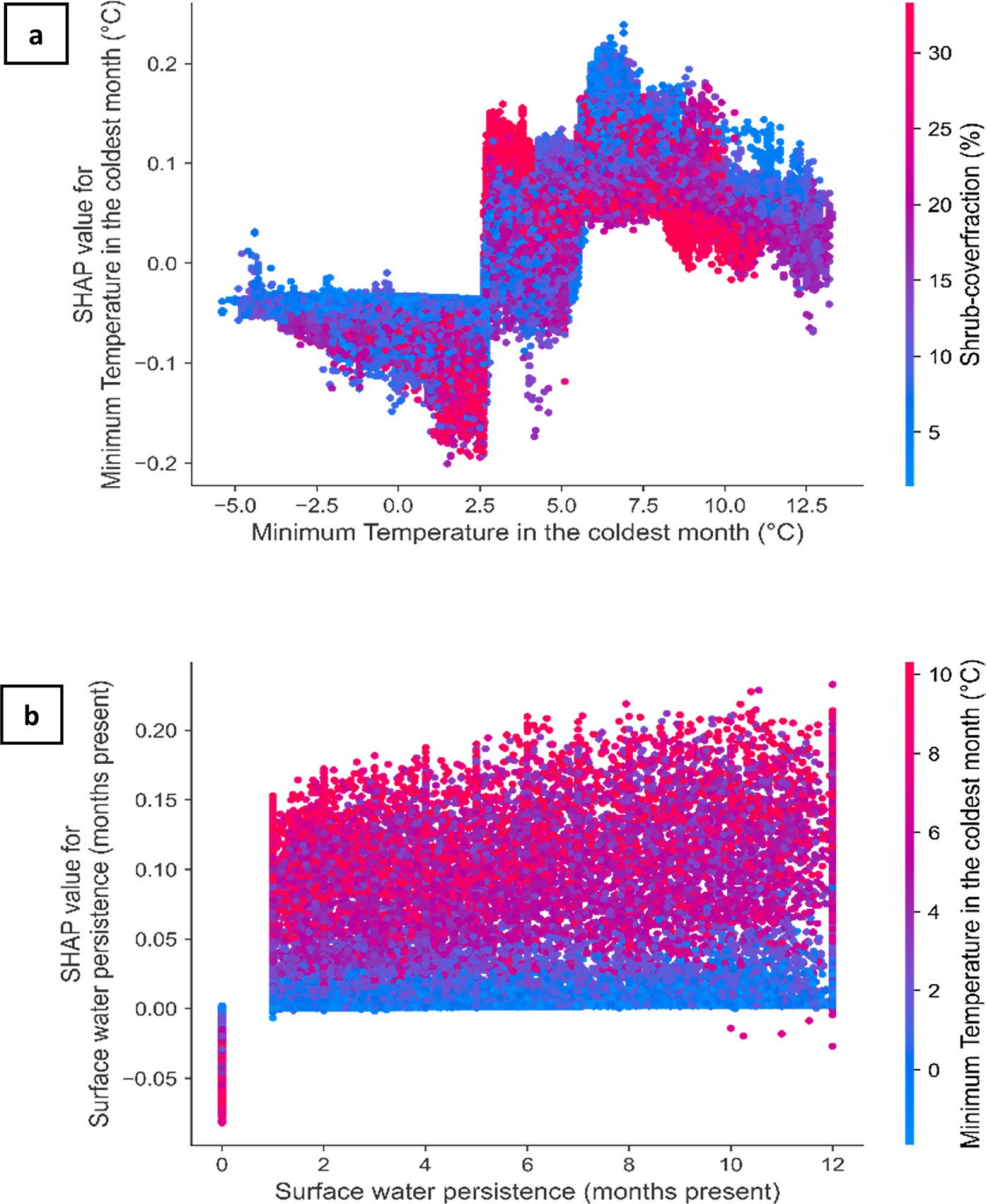
SHAP dependence plots with interaction effects illustrating the effect of the two most influential predictors of water hyacinth occurrence. (**a**) Minimum temperature in the coldest month (°C), and (**b**) Surface water persistence (months present). The x-axis shows the value of the predictor, while the y-axis indicates the SHAP value, representing the feature’s contribution to the model prediction. Positive SHAP values correspond to an increased likelihood of infestation. Each point represents a water body, colored according to the value of the feature that explains the most remaining variance (blue–red). In (**a**), abrupt shifts in SHAP values are observed around 2.5 °C and 5 °C, suggesting potential ecological thresholds. In (**b**), water bodies with high persistence (> 10 months) and warmer winter temperatures consistently show increased positive contributions compared to those with low persistence (< 2 months) and low temperatures

We applied SHAP to understand how different environmental features drive the occurrence of water hyacinth. By generating SHAP values for each instance and predictor, we gained insights into both local and global feature importance within our model. SHAP values provide three key analytical approaches in our study. Using partial dependence plots and SHAP value analyses, we evaluate how variation in each environmental feature alters the predicted suitability of a water body for water hyacinth and quantify the contribution of each feature to the overall model output (Fig. 4). For local interpretation, Roodekoppies Dam was selected as the example due to its favorable characteristics for remote sensing analysis: it is large enough to be reliably detected in medium-resolution satellite imagery and is located downstream from Hartbeespoort Dam—a known hotspot for water hyacinth invasion—within an agriculturally intensive catchment. By summing SHAP values for each feature across all water bodies, we obtain a measure of how much each feature contributes to the model’s overall predictions. This allows us to rank features by their relative importance either at the national scale (i.e., across South Africa) or within specific cohorts of water bodies (e.g., grouped by province or water body type) (Fig. 5). Features with higher absolute SHAP values have a greater contribution to predicting water hyacinth occurrence. Additionally, SHAP dependence plots enable us to interpret suitability responses to changing environmental conditions. These plots also reveal interactions among features by displaying vertical dispersion at specific feature values (Fig. 6).

We report SHAP values on a log odds scale to account for technical constraints in translating SHAP contributions to probability values. For reference, log odds of 0 correspond to a probability of approximately 0.5, while extreme values of − 5 and 5 approach probabilities of 0 and 1, respectively.

### Implementation details

All analyses were conducted in Python, using the GEE Python API (Gorelick et al., 2017). The *geemap* library (Wu, 2020) facilitated batch extraction of covariates from GEE for all water bodies. Feature selection was performed using the RFECV method from *scikit-learn*, and model selection and training were carried out with *pycaret* (Ali, 2020). Hyperparameter tuning was implemented using *Hyperopt* (Bergstra et al., 2015), and model interpretability was assessed using *SHAP* and *fastTreeSHAP* (Lundberg et al. 2020). Figures and spatial visualizations were generated using *matplotlib* (Barrett et al., 2005), *seaborn* (Waskom, 2021), *geopandas* (Jordahl, 2014), and *contextily* (Arribas-Bel, 2021).

## Results

### Model evaluation

Model accuracy is critical for ensuring the reliability and consistency of explanations across different explainable AI (xAI) methods (Liu & Udell, 2020). Higher accuracy typically correlates with better agreement among various xAI techniques. Our model exhibits good overall performance (Fig. 3), with an F1 score exceeding 0.7—a threshold commonly interpreted in the literature as indicative of strong predictive capability (Liu & Udell, 2020). However, when evaluated using the Matthews correlation coefficient (MCC), the model’s effectiveness shows a slight decrease (Fig. 3, mean MCC = 0.688). This discrepancy can be attributed to difficulties in correctly classifying uninvaded water bodies.

The MCC metric also exhibits greater variability (Fig. 3), differing from the trends observed in other metrics. Notably, model precision is the highest metric (Fig. 3, mean = 0.890), indicating the correct identification of 89.0% of water hyacinth-invaded sites. This high precision is advantageous for understanding the presence of the species rather than its absence. The high precision score, accompanied by a relatively elevated false negative rate, reflects our decision to optimize the model for precision. This minimizes false positives—i.e., incorrectly predicting infestations where none exist—which is useful when using model-derived distributional data with inherent errors. However, this comes at the cost of increased false negatives, where some actual infestations may go undetected.

### Local (per water body) interpretation

To evaluate the contributions of the final 82 selected variables to the occurrence of water hyacinth at specific sites, we used a waterfall plot (Fig. 4). At Roodekoppies Dam, with a known water hyacinth infestation, the probability of occurrence is 0.517, notably higher than the average probability of 0.114 observed across all sites in South Africa. The primary factors promoting the occurrence of water hyacinth at Roodekoppies Dam include a relatively high minimum temperature of 2.9 °C during the coldest month and the presence of surface water for more than 9 months of the year. Conversely, the precipitation level of approximately 474.21 mm—close to the national average of 463.42 mm (World bank group, 2021)—is associated with lower suitability for water hyacinth proliferation. Presumably, low rainfall results in stranded plants (Venter et al., 2017), while high rainfall causes flooding that washes out seeds and plants (J R Wilson et al., 2000). Alternatively, low runoff results in nutrient limitation (Carignan & Neiff, 1992). We use Roodekoppies Dam as a demonstrative example to illustrate the interpretability of SHAP at a local scale, given its contextual relevance and observed infestation dynamics.

### Global feature importance

Among the 82 selected features, 60 (> 72%) are associated with land use and land cover (LULC) (Fig. 5). This highlights the significant impact of surrounding land cover on the occurrence of water hyacinth. Notably, 38 of the 60 land use and land cover (LULC) features (63%) are linked to human modification. However, among all predictors considered—including climatic, topographic, and LULC variables—the most significant predictor of water hyacinth presence was a climatic variable, the minimum temperature (Fig. 5).

Our findings highlight previously underexplored predictors of water hyacinth distribution, complementing the well-documented association with low temperatures (Byrne et al., 2010; Gettys et al., 2014). Specifically, our analysis indicates that moderate shrub cover within a 5-km buffer is more significant than tree and grass cover (Fig. 5). Moreover, water systems with low topographic shading effects and heterogeneous temperature and moisture conditions—characterized by low Continuous Heat Insolation Load Index (CHILI) values and high topographic diversity—enhance the suitability for water hyacinth occurrence (Appendix, Fig. 10a and b).

### Feature dependence and interactions

SHAP dependency plots are a feature-level plot that displays points that correspond to individual sites, providing insights into how feature importance varies as feature values change. The plots also color-code points based on the feature that explains the most significant variation among the remaining modeled features. This informative visualization serves as a valuable tool for discerning species-environment responses, pinpointing inflection points indicative of abrupt alterations in feature importance, and elucidating interactions among factors potentially influencing the occurrence of water hyacinth. Noteworthy instances include sharp increases in the contribution of minimum temperature to predicted water hyacinth suitability at approximately 2.5 °C and 5 °C. These thresholds suggest that water hyacinth is more likely to occur in areas where winter temperatures remain above these values—reflecting lower temperature limits for survival or growth—highlighting the species’ sensitivity to cold stress. The inflection point situated at 2.5 °C assumes a greater degree of importance in constraining the occurrence of water hyacinth, as evidenced by SHAP values surpassing 0 at temperatures exceeding 2.5 °C. At this threshold (~ 2.5 °C), a higher shrub cover fraction is correlated with an increased likelihood of water hyacinth occurrence, showing a stronger association than observed at the latter inflection point (~ 5 °C; Fig. 6a). Furthermore, surface water persistence exhibits a gradual positive correlation with water hyacinth occurrence (Fig. 6b). The combined influence of surface water persistence (represented along the x-axis) and warmer temperatures (denoted by pink-red colors) enhances the suitability of a water system for the proliferation of water hyacinth. Water hyacinth occurrence shows an interesting non-linear response to the human modification index (Appendix, Fig. 10d), indicating a dual influence of human modification as both a facilitator and constraining factor of water hyacinth occurrence.

### Spatial distribution of features’ importance

**Fig. 7:**
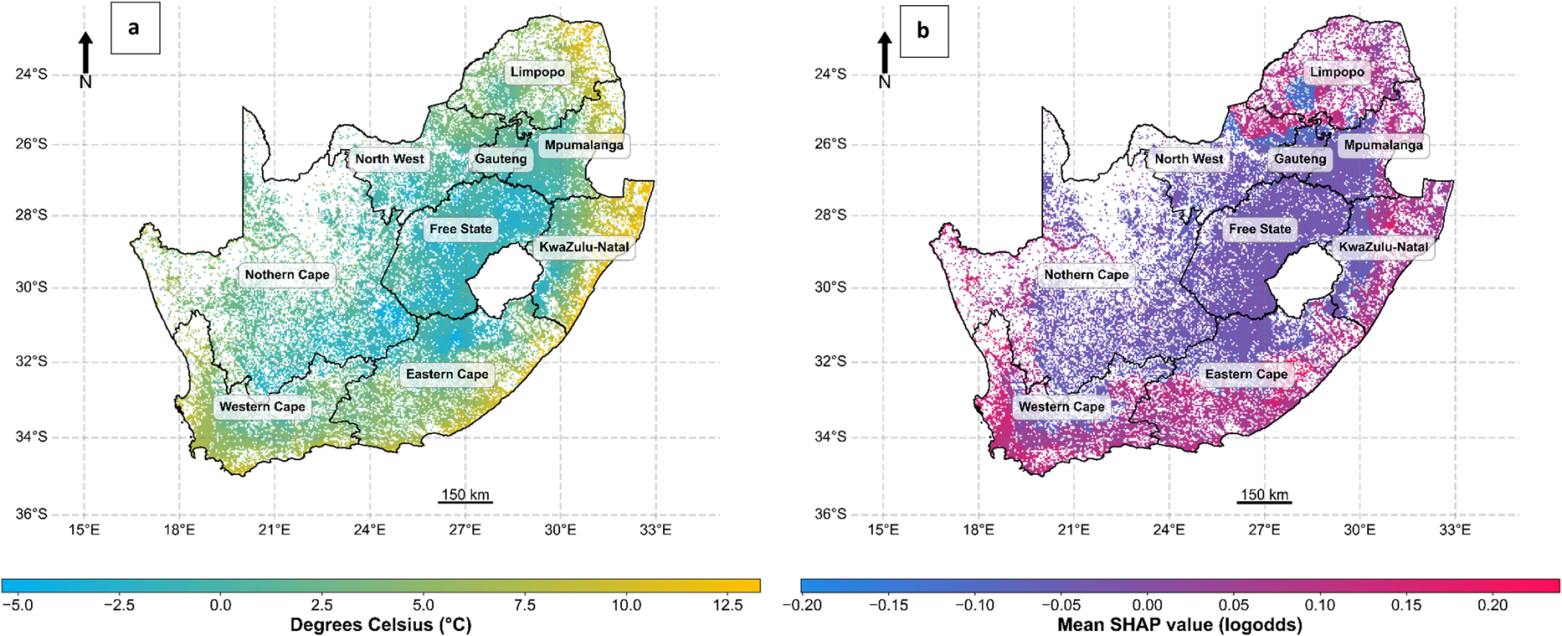
The distribution of minimum temperature (**a**)— the top national predictive feature of water hyacinth occurrence and the SHAP values for minimum temperature (**b**) across South Africa. SHAP values greater than 0 correspond to areas where less extreme minimum temperature does not hinder the occurrence of water hyacinth (limited mainly to coastal areas), while the interior of the country experiences more extreme cold temperatures, reducing the suitability for water hyacinth

To depict the spatial distribution of variable importance, the SHAP values of individual variables were aggregated at a 5-km block level (Fig. 7). This aggregation was achieved by computing the mean SHAP value of each variable within the corresponding block. Notably, lower temperatures exhibit a negative correlation with the likelihood of water hyacinth occurrence. Moreover, the log odds of water hyacinth occurrence have a much more abrupt change (Fig. 7b) in relation to the temperature gradient (Fig. 7a). The low suitability of the country’s interior, based on minimum temperature, is also evident (Fig. 7b).

**Fig. 8:**
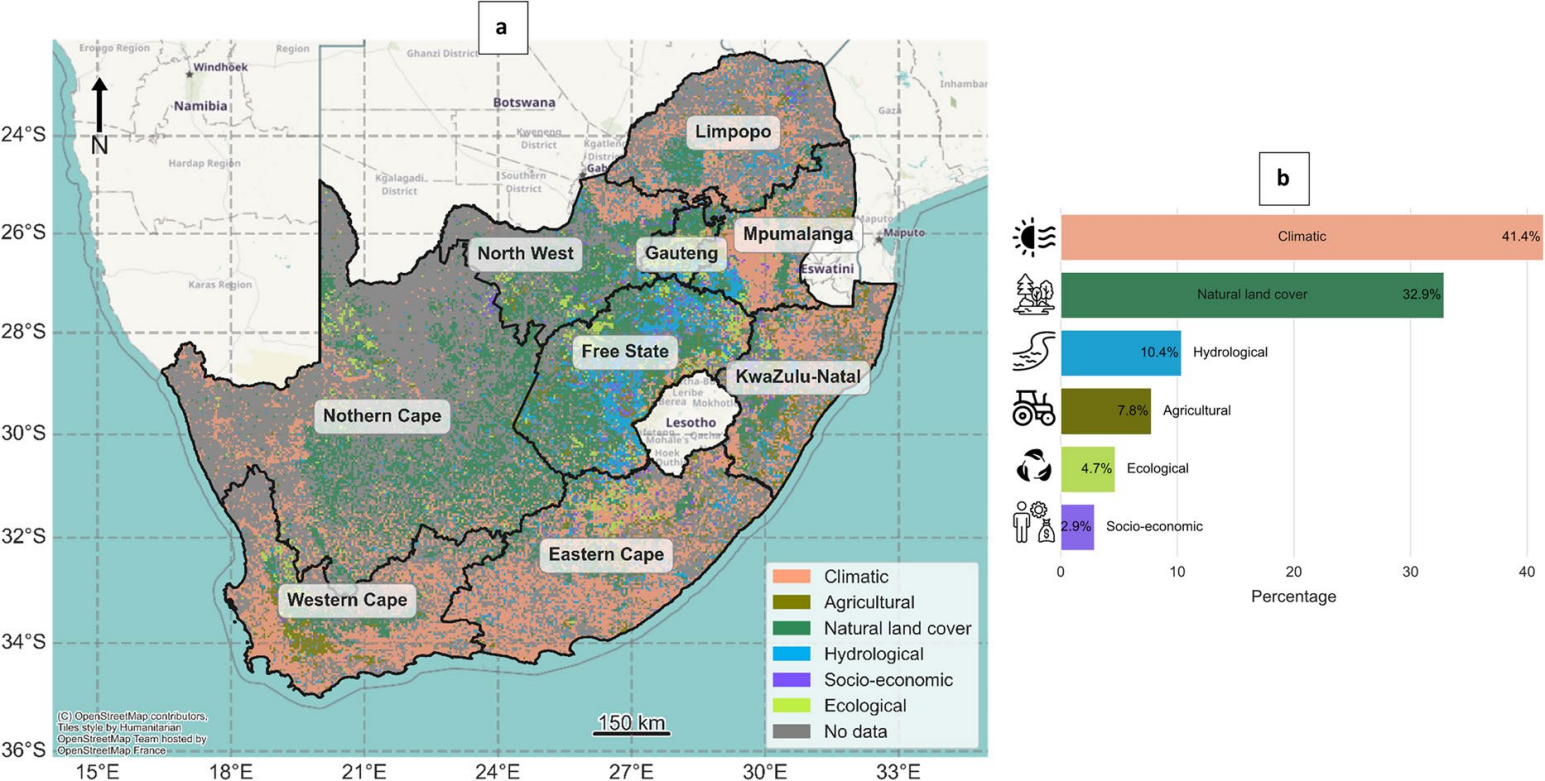
The distribution of the most important feature (feature with the highest mean SHAP value) promoting water hyacinth occurrence at South African water systems per (5 × 5 km) block across South Africa (**a**) and the proportion cover of each group (**b**). Gray areas correspond to areas of no surface water and therefore no data. For a list of the factors included in each of the six groups, refer to Table A4. Note that climatic factors dominate in the coastal regions, whereas non-climate variables dominate in the interior of the country

**Fig. 9:**
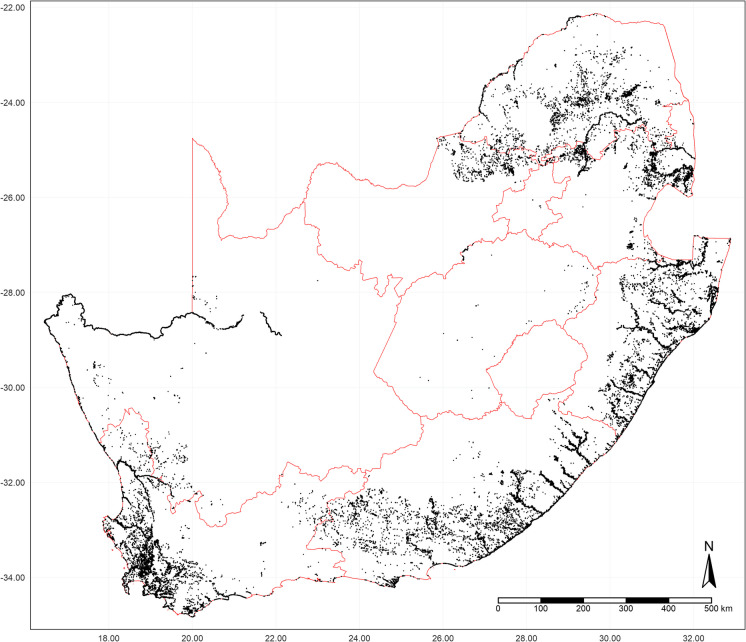
The cumulative water hyacinth distribution for 2013 across South Africa (adapted from Singh et al., 2020). The area of water hyacinth infestation is 417.7 km^2^ or 0.03% of the total area of South Africa

Warmer temperatures, as indicated by higher minimum temperature values, are correlated with predicted water hyacinth suitability in coastal regions. In contrast, natural land cover features are more strongly associated with suitability in inland areas (Fig. 8), such as the Free State, where the most influential variables include tree and shrub cover, as well as precipitation (Fig. 5). Therefore, the hydrological and natural land cover groups emerge as the predominant predictor groups shaping the occurrence patterns of water hyacinth in this region. This map does not indicate areas of model extrapolation as shown in a Multivariate Environmental Similarity Surface (MESS) map produced by Maxent (Elith et al., 2010). Nor does it correspond to the “most dissimilar variable” plot commonly generated alongside MESS outputs, which identifies the covariate contributing most to environmental dissimilarity from the training data. Instead, this map identifies, for each spatial block, the covariate group with the highest average SHAP value, thus highlighting the dominant feature group associated with water hyacinth occurrence across regions.

## Discussion

Identification of the most suitable management strategy for an IAP depends on understanding the behavior of the plant in various environmental and socio-economic contexts (John R Wilson et al., 2005). In this study, we leveraged pre-existing EO-derived datasets, SDMs, and SHAP to discern the relative importance of features that probably influence the distribution of water hyacinth in South Africa at multiple spatial scales.

Our analysis of species–environment interactions for water hyacinth aligns with the plant’s known environmental preferences, particularly its sensitivity to cold. The species rarely persists where minimum temperatures fall below 2.5 °C, with higher suitability observed above 5–8 °C—thresholds that are consistent with experimental studies and the thermal limits of associated biocontrol agents (Gopal, 1987; King, 2011; Owens & Madsen, 1995). This research offers new quantitative insights into plant–environment interactions through a nationwide, spatially explicit analysis of water hyacinth occurrence. In addition to highlighting general correlations with environmental and socio-economic variables, the significant variation in species–environment responses across provinces and sites emphasizes the value of interpretable models for guiding localized management strategies.

### The influence of climatic factors on water hyacinth distribution

#### Temperature

Temperature plays a critical role in shaping the growth, survival, and spread of water hyacinth, making it one of the most thoroughly investigated environmental variables of the species’ ecology (Gopal, 1987; Owens & Madsen, 1995; John R Wilson et al., 2005). Understanding this relationship is especially important for validating model outputs. In this study, we examined temperature ranges with established physiological significance to interpret changes in the log-odds of water hyacinth occurrence, as revealed by SHAP values. These SHAP contributions align well with known biological responses. For instance, air temperatures below 0 °C are lethal to the plant’s above-water parts (Madsen et al., 1993), and survival during winter becomes highly unlikely when minimum temperatures fall between 0 and 2.5 °C. This is reflected in our model by strongly negative SHAP values and a sharp decline in predicted occurrence below the 2.5 °C threshold. Such alignment enhances the ecological interpretability of the model and supports the reliability of SHAP-derived inferences. While low temperatures limit the plant’s broad distribution, microclimatic refugia may permit localized overwintering even in areas below this threshold, accounting for the occurrence of water hyacinth in areas with minimum temperatures below 2.5 °C (Kriticos & Brunel, 2016; Miskella & Madsen, 2019).

Positive SHAP values in the range of 2.5 to 5 °C show the potential persistence of water hyacinth. However, these sites will harbor small populations, as short periods of cold exposure (below 5 °C for less than 2 weeks) may not kill the plant (Owens & Madsen, 1995). The survival of water hyacinth at these low temperatures may also be due to the reduced effectiveness of biological control agents under such conditions (J A Coetzee et al., 2011). Low temperatures and subsequent diminished plant quantity and quality may hinder the establishment of biological control agents or suppress their population growth by affecting their developmental processes (Byrne et al., 2010; M P Hill & Cilliers, 1999; M P Hill & Olckers, 2000). Between 5 and 8 °C, there is a significant increase in the suitability for water hyacinth, indicating enhanced suitability under warmer temperatures. Beyond this range, we expect warmer temperatures to continue to improve the suitability for water hyacinth; however, a gradual decrease in the log odds of water hyacinth presence is observed. This may be due to the increased management attention that thriving populations receive and the improved efficacy of biocontrol agents in warmer climates. Since these ranges and thresholds align with known ecological thresholds and physiological tolerances, there is increased support for SHAP-based model explanations and suitability to help direct management resources to areas that maximize biocontrol success.

Warmer temperatures (> 8 °C) with positive SHAP values, indicative of promoting water hyacinth occurrence, are close to known controlled laboratory-determined lower thermal developmental thresholds of biocontrol agents (e.g., 10–15 °C for two weevil biocontrol agents) (Julien, 2000). Where temperatures exceed 8 °C, biocontrol agents have a higher chance of survival and efficacy. Subsequently, areas with prolonged minimum temperatures below 8 °C could focus less on extensive biocontrol investments and more on mechanical or chemical methods, given the limited likelihood of plant persistence or effective biocontrol in colder regions. Therefore, SHAP can inform managers on where to allocate biocontrol resources, focusing on areas where models predict high water hyacinth suitability due to warmer temperatures. This data-driven approach aligns biological control efforts with ecological feasibility, enhancing intervention effectiveness.

#### Microclimate effects

For IAP management, SHAP can assist in identifying areas that are most at risk by quantifying the impact of interaction effects between variables, for example, topographic diversity and urban proximity on model predictions. Topographic diversity, which reflects local moisture and temperature conditions, is positively associated with a variety of temperature-moisture habitats. Increased topographic diversity has been linked to greater species resilience under climate change (Lawrence et al., 2020). The response of water hyacinth suitability to both topographic diversity and urban area suggests that uninvaded water bodies surrounded by landscapes with high topographic diversity (> ~ 0.5) and substantial urban area (2.5–3 km^2^) within a 5-km radius may be particularly vulnerable to invasion. Consequently, any water body with a high predicted suitability owing to dominant contributions from the urban area and topographic diversity features could be automatically assigned a raised early-detection priority, allowing managers to rank and schedule monitoring efforts objectively. This makes SHAP an effective tool for prioritizing monitoring efforts, especially in vulnerable habitats (M P Hill, 2003; VonBank et al., 2018).

#### Precipitation and surface water persistence

**Fig. 10:**
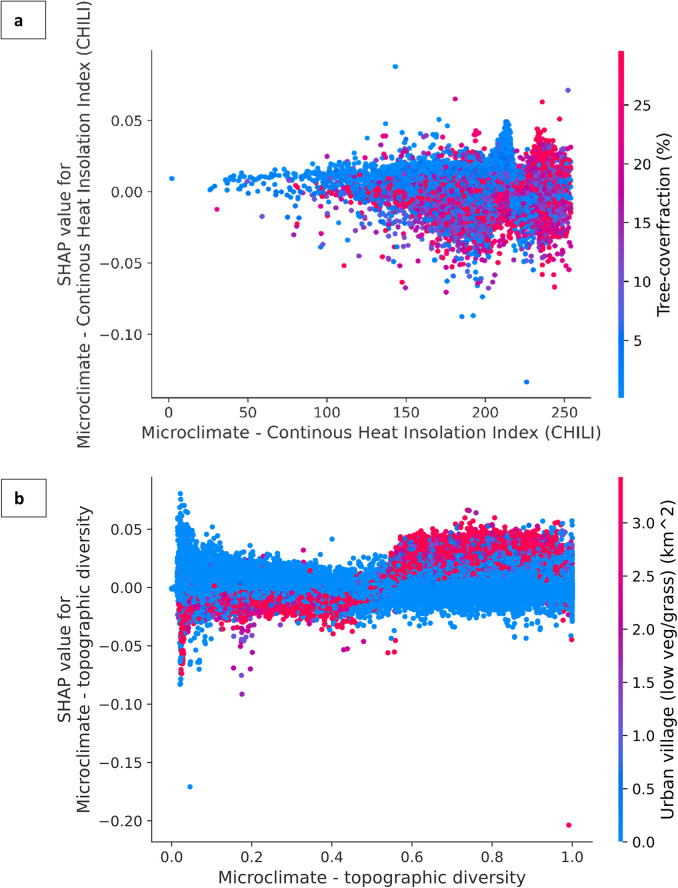

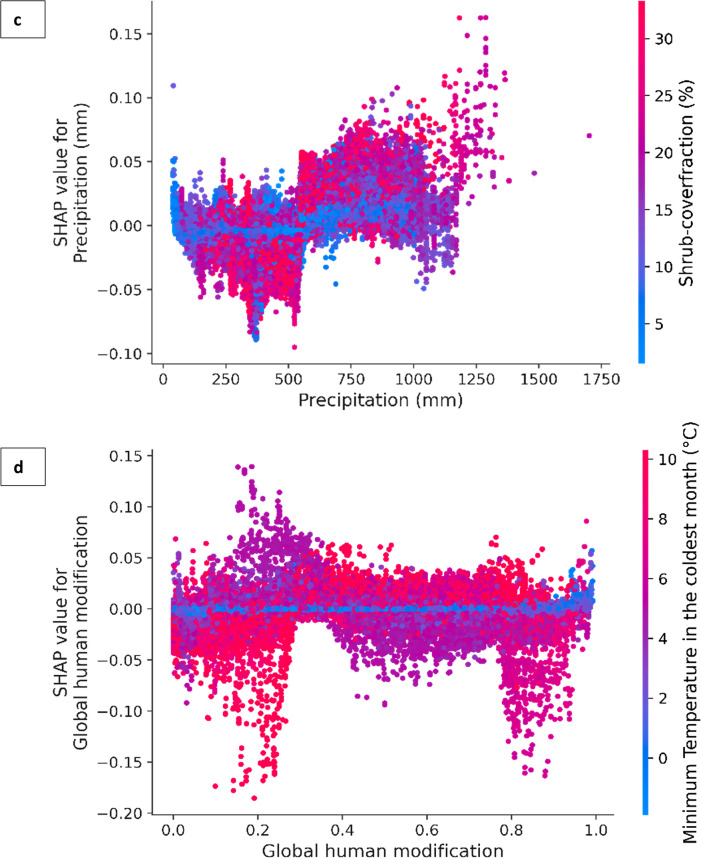

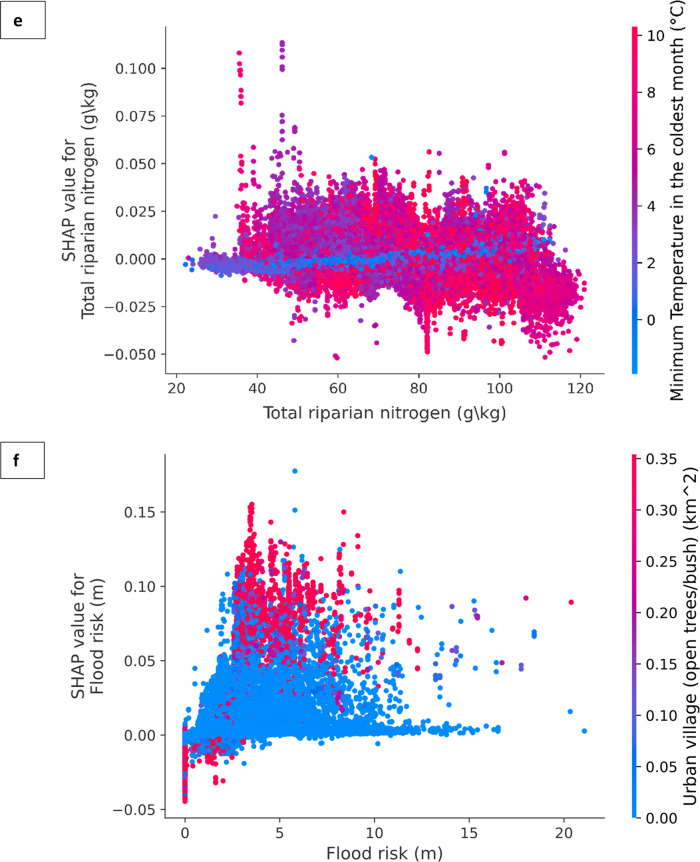
SHAP dependence plots with interaction effects for (**a**) Continuous Heat Insolation Index (CHILI) and (**b**) topographic diversity – a proxy for topo-climate niches and micro-climate. Also includes feature interactions indicated by the blue-red colour. SHAP dependence plots with interaction effects for (**c**) total precipitation (mm) for 2013, (**d**) global human modification— an index that reflects anthropogenic influences and landscape alteration. Also includes feature interactions indicated by the blue-red colour. SHAP dependence plots with interaction effects for (**e**) Riparian total soil nitrogen (–/kg)— a proxy of water nitrogen levels (**f**) 10-year return flood hazard (metres). Also includes feature interactions indicated by the blue-red colour. Sites with high urban area are associated with a greater risk of water hyacinth

SHAP dependence plots can illustrate precipitation thresholds above which the likelihood of water hyacinth increases (e.g., the > 560-mm per year threshold, Appendix, Fig. 10c). A resurgence of water hyacinth at New Year’s Dam, a small (150 ha), shallow, oligotrophic dam in the Eastern Cape in 1998 has been linked with above-average rainfall for this semi-arid region (> 350–550 mm Palmer, 2004; Zengeni et al., 2016)) (M P Hill & Olckers, 2000). The dependence plot for precipitation indicates that annual accumulated precipitation exceeding ~ 560 mm (above the national average of 463 mm (World bank group, 2021)) correlates with a higher probability of water hyacinth presence, supporting the role of increased rainfall in the water hyacinth resurgence event at New Year’s Dam.

Precipitation directly influences surface water presence and persistence, with a positive relationship evident between water persistence and water hyacinth occurrence (Fig. 6b). Although water hyacinth can adapt to varying water levels (Venter et al., 2017), permanent water bodies are much more susceptible to invasion than seasonal ones, likely due to the stability they offer (Fig. 6b). Permanent water bodies have 2–3 times the (log) odds of supporting water hyacinth compared to seasonal ones (present for 1–3 months of 2013). For example, in the dry Karoo areas of the Northern Cape, the limited number of permanent water bodies restricts water hyacinth growth despite suitable temperatures (Fig. 8a). This insight is useful for managers to prioritize high-persistence or permanent water bodies for ongoing monitoring, where invasions are most likely.

#### Socio-economic effects

SHAP can be instrumental in quantifying the dual role of human activities within predictive models, offering insights into how human-driven factors facilitate or hinder invasive species like water hyacinth in a non-linear manner. The presence and spread of alien and invasive species are strongly linked to human-assisted dispersal and introduction (M P Hill, 2003), as well as human-induced disturbances facilitating establishment (VonBank et al., 2018). Using a global human modification metric that integrates data from 13 datasets on human settlement, agriculture, transportation, mining, and energy production (Kennedy et al., 2019), we observe a parabolic relationship with the presence of water hyacinth (Appendix, Fig. 10d). This parabolic relationship, where risk peaks around 25% human modification, can guide managers to focus on areas with moderate human disturbance for prevention and monitoring. Areas below this threshold can also be deprioritized in terms of risk. Higher levels of human modification (> 25%) may result in disturbances that exceed the tolerance levels of water hyacinth or lead to active removal efforts in urban and agricultural areas (cultivated subsistence or cultivated orchards, Fig. 5). Additionally, poorly functioning wastewater treatment facilities contribute to eutrophic water conditions in South Africa, exacerbating the problem (Harding, 2015; Oberholster & Ashton, 2008). Overall, SHAP’s role in revealing the nuanced relationship between human modification and IAP occurrence equips managers with actionable insights, enabling them to adapt interventions based on specific local conditions and modify land-use policies or remediation efforts to support IAP management.

#### Ecological effects

The role of shrub cover in moderating nutrient run-off illustrates how certain land features can reduce or amplify invasion risk depending on temperature and rainfall. SHAP interaction effects revealed a temperature-dependent relationship between shrub cover and water hyacinth invasion suitability, where shrub cover functions as a thermal moderator with opposing effects across different climatic contexts. Within the 2.5 to 4 °C minimum temperature range, high shrub cover (> 25%) increases suitability by buffering cold winter temperatures and enhancing overwintering survival (Dugdale et al., 2018), while at all other temperatures, shrub cover reduces suitability through mechanisms such as nutrient buffering, increased shading, or surface water cooling. This context-dependent reversal demonstrates that the same landscape feature can either facilitate or inhibit invasion depending on baseline thermal conditions, highlighting the importance of spatially nuanced interpretations of variable interactions in invasion ecology.

At Roodekoppies Dam, slightly above-average rainfall (474 mm vs. the national average 463 mm) combined with high shrub cover (> 25%, with 27.8% at Roodekoppies Dam), within the 5-km riparian zone generally reduces the suitability for water hyacinth. This may be due to the buffering effect of riparian shrubs, which limit nutrient runoff into water systems (Aguiar Jr et al. 2015; Jiang et al., 2020). However, during periods of high rainfall (575–600 mm) and accelerated runoff, the buffering effect diminishes, increasing nutrient release into adjacent waters, especially in nitrogen-saturated agricultural soils (Jiang et al., 2020; Sabater et al., 2003; Taylor & Townsend, 2010). By capturing the impact of fluctuating rainfall on IAP risk, SHAP can guide adaptive management practices. For example, during periods of high rainfall, efforts to mitigate nutrient runoff or establish physical barriers might be prioritized for vulnerable water bodies near nitrogen-rich agricultural soils.

Interestingly, we also found that shrub cover has stronger interaction effects with minimum temperature, the strongest predictor of water hyacinth occurrence, compared to tree or grass cover. This could be attributed to various factors such as tree cover distribution, land use effects, and riparian buffer width (Aguiar Jr et al. 2015; Jiang et al., 2020; Sabater et al., 2003; Taylor & Townsend, 2010). First, grass cover is considered the least important predictor of water hyacinth occurrence among the three features; this is likely because the shrub and tree cover predictive features both encompass non-agricultural and agricultural vegetation— a known contributor to high-nutrient runoff. Woody perennial crops under 5 m tall are included in shrub cover, while those over 5 m fall under tree cover. Next, shrub cover is likely a stronger predictor than tree cover owing to its more effective nutrient buffering abilities (Aguiar Jr et al. 2015; Cole et al., 2020). Moreover, the Western Cape, with more water hyacinth but lower tree cover, suggests that tree cover is not as generalizable a predictor of water hyacinth compared to shrub cover across large extents.

Comprehensive satellite or field-based water nutrient level estimates are unavailable for South Africa and are challenging to estimate from satellite imagery (Schaeffer et al., 2013; Silberbauer, 2020; Slaughter et al., 2017). Thus, soil nutrients, runoff, and agricultural land cover variables for the 5-km area surrounding a water body were used as a proxy (Sharpley et al., 2003). Eutrophic water conditions drive water hyacinth invasions and are expected to increase with adjacent soil nutrient runoff (Bick et al., 2020). However, contrary to existing research, total riparian soil nitrogen had an inconsistent effect on water hyacinth occurrence, suggesting it is an inadequate proxy for water nitrogen content (Appendix, Fig. 10e). In contrast, both agricultural and urban land cover were indicative of increased water hyacinth suitability, suggesting their promise as good proxies of water nutrients (Fig. 5). The limited utility of the 5-km riparian soil nutrients as proxies for water nutrient levels may be due to differences in the biogeochemical processes in the actual riparian zone (typically 10–100 m) and the 5-km buffer zone around the water body that was considered (Bredin & Macfarlane, 2017). Therefore, future studies should consider multiple buffer widths and additional water nitrogen proxies, such as variables that capture the intensity of agriculture or the compliance of wastewater treatment facilities. From the variables considered in this study, the area of the urban class and soil organic carbon showed the highest correlation (~ 0.4) with in situ water nitrogen data for 2013, making them priority candidates for water quality proxies.

Floods are associated with an increased chance of water hyacinth occurrence, especially with the presence of surrounding urban land use (Pérez et al., 2011a, 2011b) (Appendix, Fig. 10f). Floods enhance dispersal, allow germination of buried seeds in open water, and increase nutrient inflow (Neiff et al., 2001; Pérez et al., 2011a, 2011b). They may also reduce biocontrol effectiveness as biocontrol agent populations take longer to recover than their host plant (Cilliers, 1991). However, in near-coastal areas, floods may force plants into saline conditions intolerable for water hyacinth (Coetzee et al., 2017). The results suggest that floods predominantly act as a facilitator of water hyacinth invasion, and as a regulator of water hyacinth populations at a much smaller subset of sites during 2013 (Appendix, Fig. 10f). This knowledge aids managers in deprioritizing costly interventions in regions where natural salinity or flood-prone zones will mitigate spread.

#### Benefits and drawbacks

Correlative SDMs are valuable for mapping and managing the risk of invasive alien aquatic plant (IAAP) species. However, they are often criticized for lacking a biological basis (Srivastava et al., 2019). To address this, modeling experts incorporate prior knowledge of species’ requirements and tolerances to select relevant variables for modeling. Despite these efforts, the correlative nature of SDMs, such as those used in this study, necessitates cautious interpretation, particularly when species-environment feedback mechanisms are involved. For instance, floods can flush out nuisance water hyacinth populations. Simultaneously, water hyacinth mats can increase the risk of floods by reducing stream flow (Neiff et al., 2001).

Using EO-derived distribution data offers advantages over presence-background data used in Maxent or Ecological Niche Factor Analyses (ENFAs) and presence/pseudo-absence data used in other machine learning algorithms (Chapman et al., 2019). EO-derived data provide reduced uncertainty compared to background or pseudo-absence data and are less susceptible to sample bias compared to costly field-collected samples, which can violate the assumption of independence among species records (Guillera‐Arroita, 2017, Chapman et al., 2019). Consequently, the output habitat suitability maps may correspond not only to the species’ observed distribution but also to the distribution of sampling effort. However, EO-derived distributions can still suffer from mapping errors, including residual spatial autocorrelation, which affects the reliability of SDMs. However, by combining SHAP with a block cross-validation strategy and optimizing for the precision metric, we reduced the effect of these errors on the models’ interpretation.

In this study, the SDM showed an error of less than 20% based on the F1-score. This error may be due to suitable environmental conditions for water hyacinth where it has not yet been introduced, suggesting dispersal constraints and a lack of equilibrium with the environment in South Africa (Normand et al., 2011). Additionally, the spatially varying accuracy of the datasets used and the omission of relevant variables such as turbidity, water nutrient levels, management history, and water depth may contribute to the error (Venter et al., 2017).

## Conclusion

This study highlights the geographically variable drivers of water hyacinth occurrence, suggesting that effective management efforts must be context dependent. Our findings demonstrate that EO-based input data coupled with cloud computing (GEE) and recent xAI tools represent a low-cost approach to understanding the factors that limit and promote the establishment of water hyacinth in a data-driven manner. This information can assist in the pre-selection and prioritization of management strategies on a site-by-site basis. Owing to the negligible costs of carrying out this analysis, in comparison to similar large-scale field studies, we encourage the development of EO-derived species distribution products that were foundational to these analyses. Similar analyses may inform traditional lab-controlled and artificial outdoor experiments for a variety of IAPs under weed management.

## Data Availability

The datasets generated during and/or analysed during the current study are available in the Zenodo repository, https://zenodo.org/records/14280777. All datasets used to derive this dataset (Table A1) are available publicly from the core Google Earth Engine catalogue or the Google Earth Engine community catalogue.

## Appendix

**Table A1.** Feature descriptions, associated units, temporal coverages, and data sources of the features considered to investigate the likely drivers of water hyacinth occurrence. All explanatory variables were downloaded at a 30 m spatial resolution. Those variables that were only available at a coarser spatial resolution (> 30 m) were automatically resampled to 30 m using bilinear interpolation within Google Earth Engine

Variable	Units	Spatial resolution (m)	Temporal coverage	Source
Number of consecutive nights with less than 10 degrees Celsius	days	1000	2013	MODIS LST- night
Area of water body	m^2^	30	2013	(Singh et al., 2020)
Median river width at mean discharge	m	N/A vector	1984–2018	Global River Width from Landsat (GRWL) (Allen & Pavelsky, 2018)
^a^ Area of aquatic vegetation	m^2^	30	2013	(Singh et al., 2020)
^a^ Minimum Temperature in the coldest month between 1970 and1990	°C	1000	1970–2000	WorldClim
Connectivity- upstream and downstream river count	river count	N/A vector	2000	WWF hydrosheds (Grill et al., 2019)
^a^ Surface water persistence- number of months water is present	month count	30	^b^ 1984–2019 (2013)	JRC GSW (Pekel et al., 2016)
*Total precipitation	mm	4638.3	^b^ 1958–2021 (2013)	TerraClimate
^a^ Distance to nearest coastline	m	30	2009	NOAA (https://oceancolor.gsfc.nasa.gov/docs/distfromcoast/)
*Distance to roads	m	30	1979–2010	Global Road Infrastructure Project (GRIP) (Meijer et al., 2018)
Elevation	m	30	2000	NASADEM
^a^ Continuous Heat Insolation Load Index (0 = cool, 255 = warm). Surrogate for effects of shading and topographic insolation	0–255	90	2006–2011	SRTM, (Theobald et al., 2015)
^a^ Global Human Modification (1 = high modification)	0—1	1000	2016	(Kennedy et al., 2019)
^a^ Topographic diversity- Surrogate for the variety of temperature and moisture conditions available to species as local habitats	-1323—8.81	270	2006–2011	SRTM, (Theobald et al., 2015)
Frost duration—Median duration of frost	day count	1700	2007	(Schulze & Maharaj, 2007)
Number of days below 0 °C (0–365)	day count	1000	^b^ 2000 – present (2013)	MODIS- LST, night temperature
Number of days below 10 °C (0–365)	day count	1000	^b^ 2000 – present (2013)	MODIS- LST, night temperature
^a^ South African National Land Cover (73 class)	km^2^	30	2013–2014	GeoTerraimage
^a^ Mean Annual Runoff	mm/year	~ 1852	2005	Strategic Water Source Areas (SWSA)
^a^ Broad (10) class Landcover	% (cover fraction)	100	2015	Copernicus Global Land Landcover Layers (CGLS-100)(Buchhorn et al., 2020)
^a^ Flood hazard with a 10-year return period	water depth (m)	1000	2016	Joint Research Commission (Dottori et al., 2016)
Riparian soil nitrogen (5 km buffer)	g/kg	250	1905–2016	Soil grids (Poggio et al., 2021)
Riparian soil ph (5 km buffer)	pH	250	1905–2016	Soil grids (Poggio et al., 2021)
^a^ Riparian soil carbon (5 km buffer)	g/kg	250	1905–2016	Soil grids (Poggio et al., 2021)
^a^ Stream Power Index (SPI)	-1—1	90	1987–2017	GeoMorpho90 GeoMorphometric layers (Amatulli et al., 2020)
^a^ Relative wealth index	-1—1	2400	2001–2019	Facebook (https://dataforgood.fb.com/tools/relative-wealth-index/)
13 iSDA Soil layers (includes *total nitrogen, etc.)	various	30	2013–2019	(Hengl et al., 2021)
^a^ Mean wind speed for a 10-year period	m/s	250	2008–2017	Global wind atlas
^a^ Included in final model, ^b^ Multi-temporal data available. The period for the selected data used in this study. 2013 or temporally closest data was selected				

**Table A2.** The drivers of water hyacinth considered as predictive features during modeling and documented associations

Category	Driver	Documented association (proxy)
Climatic	Temperature	(Byrne et al., 2010; Miskella & Madsen, 2019; Owens & Madsen, 1995)
	Precipitation	(Bayu et al., 2024)
	Frost	(Byrne et al., 2010)
	Wind	(John R Wilson et al., 2005)
	Topo-climatic	(Lawrence et al., 2020; Miskella & Madsen, 2019)
Socio- economic	Artificial land cover	(Essl et al., 2019)
	Human modification	(M P Hill, 2003; VonBank et al., 2018; Westphal et al., 2008)
	Reltive wealth index	Development ((Essl et al., 2019)
Ecological	Natural land cover	Riparian buffer effects (Cole et al., 2020; Jiang et al., 2020)
	Interspecies competition	(Agami & Reddy, 1990; Gopal, 1987)
	Distance to coastline	(Bick et al., 2020; Julie A Coetzee et al., 2017; Muramoto et al., 1991)
	Runoff	Nutrient input (Reddy et al., 1990)
	Flood risk	(Bick et al., 2020; Julie A Coetzee et al., 2017)
	Riparian soil nutrients	Water nutrients Riparian soil nutrients (Reddy et al., 1990)
Topographic	Elevation	(Kriticos & Brunel, 2016; Lawrence et al., 2020)
Hydrologic	River connectivity	Dispersal (Pérez et al., 2011a, 2011b)
	Stream power	Dispersal (Pérez et al., 2011a, 2011b)
	Water seasonality	(Gopal, 1987; Venter et al., 2017; John R Wilson et al., 2005)

**Table A3.** Evaluation metrics for 15 candidate models calculated based on a tenfold block cross-validation strategy and sorted by precision. The performance scores of the models are reported after feature selection but prior to hyperparameter tuning. The highest score for each metric is in bold font (refer to Methods section for metric descriptions)

Model	Recall	Prec	F1	MCC	Balanced accuracy
Random Forest Classifier	0.5823	**0.8269**	0.6831	0.6621	0.7832
Extra Trees Classifier	0.5827	0.8182	0.6801	0.6580	0.7829
CatBoost Classifier	**0.6252**	0.7906	**0.6981**	**0.6696**	**0.8018**
Gradient Boosting Classifier	0.4338	0.7737	0.5555	0.5417	0.7086
Extreme Gradient Boosting	0.6100	0.7725	0.6815	0.6511	0.7933
Light Gradient Boosting Machine	0.5762	0.7660	0.6573	0.6276	0.7766
Ridge Classifier	0.1760	0.6941	0.2798	0.3134	0.5828
Ada Boost Classifier	0.4433	0.6757	0.5351	0.5017	0.7077
Logistic Regression	0.3094	0.6272	0.4136	0.3930	0.6426
Decision Tree Classifier	0.6127	0.6009	0.6065	0.5547	0.7798
Linear Discriminant Analysis	0.3869	0.5979	0.4691	0.4286	0.6764
SVM—Linear Kernel	0.4365	0.4695	03901	0.3464	0.6662
K Neighbours Classifier	0.2698	0.4636	0.3409	0.2923	0.6145
Quadratic Discriminant Analysis	0.5887	0.4151	0.4865	0.4148	0.7402
Naive Bayes	0.5244	0.3852	0.4438	0.3643	0.7075

**Table A4.** The 82 (of 140) features used in the final Species Distribution Model. The variables have been grouped into five categories referred to in Fig. 8. A large portion of the variables relates to area of a land use and/or land cover within a 5 km buffer surrounding a water body (km^2^)

Group	Features (82 significant predictors of water hyacinth occurrence)
Hydrological	• Downstream river count • Surface water persistence (up to 12 months) • Percentage and area of permanent water area within a 5 km buffer • Percentage and area of seasonal water area within a 5 km buffer • Stream power index • Risk of flood (metres of flood water) • Runoff (mm) • Natural surface water (pans flooded at observation time)
Natural land cover	• Percentage grass cover • Percentage shrub cover • Percentage tree cover • Percentage bare cover • Forest (woodland and indigenous forest) • Shrubland (shrubland fynbos, low shrubland, thicket) • Grassland (sparsely wooded grassland)
Climatic	• Minimum temperature • Precipitation • Wind speed • CHILI (microclimate, see text for explanation) • Topographic diversity (microclimate, see text for explanation)
Ecological	• Interspecies competition (area of non-water hyacinth aquatic vegetation) • Riparian soil (organic carbon, nitrogen content and cation exchange capacity) • Riparian stone, sand and clay content • Riparian soil fertility capability classification • Erosion • Fertility capability classification • Bare non-vegetated
Socio-economic	• Plantation forest • Global human modification (see text for explanation) • Mines • Residential • Urban (smallholding, built-up, schools and sp0rts ground, township) • Industrial • Commercial • Urban cover within a 5 km buffer • Distance to roads
Agricultural	• Agriculture (subsistence, commercial, cane, orchards, vines and crop cover fraction)

## Code availability

The code for this study is made available at this GitHub repository, https://github.com/Geethen/PhD--EO_and_ML_for_water_hyacinth_management/tree/main/Chapter_2.

## Declarations

### Declarations

All authors have read, understood, and have complied as applicable with the statement on “Ethical responsibilities of Authors” as found in the Instructions for Authors.
